# Autophagy Protects From Uremic Vascular Media Calcification

**DOI:** 10.3389/fimmu.2018.01866

**Published:** 2018-08-14

**Authors:** Bianca Frauscher, Alexander H. Kirsch, Corinna Schabhüttl, Kerstin Schweighofer, Máté Kétszeri, Marion Pollheimer, Duska Dragun, Katrin Schröder, Alexander R. Rosenkranz, Kathrin Eller, Philipp Eller

**Affiliations:** ^1^Clinical Division of Nephrology, Department of Internal Medicine, Medical University of Graz, Graz, Austria; ^2^Institute of Pathology, Medical University of Graz, Graz, Austria; ^3^Clinic for Nephrology and Critical Care Medicine, Charité - Universitätsmedizin Berlin, Freie Universitat Berlin, Humboldt-Universitat zu Berlin, Berlin, Germany; ^4^Institute of Health (BIH), Berlin, Germany; ^5^Institute for Cardiovascular Physiology, Goethe-University, Frankfurt, Germany; ^6^Intensive Care Unit, Department of Internal Medicine, Medical University of Graz, Graz, Austria

**Keywords:** vascular smooth muscle cells, rapamycin, hydroxyapatite crystals, inflammation, phosphate, chronic kidney disease

## Abstract

Chronic kidney disease and diabetes mellitus are associated with extensive media calcification, which leads to increased cardiovascular morbidity and mortality. Here, we investigated the role of autophagy in the pathogenesis of uremic vascular media calcification. DBA/2 mice were fed with high-phosphate diet (HPD) in order to cause vascular calcification. DBA/2 mice on standard chow diet were used as control. In parallel, autophagy and its response to rapamycin, 3-methyladenine (3-MA), and bafilomycin were studied in an *in vitro* model using mouse vascular smooth muscle cells (MOVAS). DBA/2 mice on HPD developed severe vascular media calcification, which is mirrored *in vitro* by culturing MOVAS under calcifying conditions. Both, *in vitro* and *in vivo*, autophagy significantly increased in MOVAS under calcifying conditions and in aortas of HPD mice, respectively. Histologically, autophagy was located to the aortic *Tunica media*, but also vascular endothelial cells, and was found to continuously increase during HPD treatment. 3-MA as well as bafilomycin blocked autophagy in MOVAS and increased calcification. *Vice versa*, rapamycin treatment further increased autophagy and resulted in a significant decrease of vascular calcification *in vitro* and *in vivo*. Rapamycin reduced *Runx2* transcription levels in aortas and MOVAS to control levels, whereas it increased α-smooth muscle actin and *Sm22α* transcription in MOVAS to control levels. Furthermore, rapamycin-treated HPD mice survived significantly longer compared to HPD controls. These findings indicate that autophagy is an endogenous response of vascular smooth muscle cells (VSMC) to protect from calcification in uremia. Induction of autophagy by rapamycin protects cells and mice from uremic media calcification possibly by inhibiting osteogenic transdifferentiation of VSMC.

## Introduction

Chronic kidney disease (CKD) and especially end stage renal disease (ESRD) are associated with an extensive increase in cardiovascular mortality and morbidity (1). Cardiovascular disease is the single greatest cause of mortality in CKD/ESRD, and it is to a large extent driven by abnormal mineral metabolism leading to extensive arterial calcifications, a reduced vascular compliance, left ventricular hypertrophy, and sudden cardiac death (2). Contrary to non-uremic patients where arterial calcification typically affects intimal atherosclerotic plaques, patients with CKD predominantly develop calcification of the tunica media (3, 4). It is currently believed that increase in serum phosphate levels is the driving force leading to vascular media calcification (5). High-phosphate levels in combination with other mediators, which are dysregulated in uremia, induce vascular smooth muscle cells (VSMC) to transdifferentiate from a contractile to proliferative, osteoblastic, and/or inflammatory phenotypes (6, 7). Interestingly, the adverse effects of high serum phosphate and/or phosphate overload in human health do not seem to be limited to advanced stages of CKD, as it has been found in earlier stages of CKD and also in the general population (8–10).

Different murine models of CKD mimicking media calcification exist (11). Recently, we established a new murine model with acute renal failure due to phosphate nephropathy which can be induced in DBA/2 mice subjected to high-phosphate diet (HPD) (12–14). DBA/2 mice have an alternative splice variant of the *Abcc6* gene resulting in an increased susceptibility to develop tissue calcification (15). Within 5–14 days mice develop calcification within the *Tunica media* of the aorta, which is more pronounced in the abdominal part of the aorta mimicking the situation in ESRD patients (14). Whereas inflammation is of crucial importance in the development of renal calcification (13), vascular media calcification does not seem to be dependent on immune cells (14).

The three different types of autophagy, namely microautophagy, chaperone-mediated autophagy, and macroautophagy, have all been extensively reviewed in the past (16, 17). We focused in our studies on macroautophagy, which will be referred to as autophagy hereafter. Autophagy is a highly conserved cellular process responsible for removal or recycling of long-lived proteins and organelles. It is essential for cell survival, differentiation, and development, and the cellular response to stress (18). A widely used marker of autophagy is microtubule-associated protein light chain 3 (LC3). It exists as cytosolic LC3-I and converts to LC3-II, which mainly inserts into isolation membranes and autophagosomes. The amount of LC3-II closely correlates with the number of autophagosomes (19). The p62 protein, also called sequestosome 1, is a ubiquitin-binding scaffold protein, which is degraded by autophagy (20). Thereby low levels of p62 protein represent states of augmented autophagy.

Since autophagy takes place in every eukaryotic cell, it plays an important role in all organ systems and has been implicated in an expanding list of disease processes (16). Growing evidence suggests that autophagy also plays a role in vascular pathophysiology including uremic media calcification (21). Dai and coworkers provided *in vitro* evidence that high-phosphate levels induced radical oxygen species (ROS) production results in increased autophagy in VSMC. This process protected cells from calcification thereby providing an endogenous protective mechanism counteracting phosphate-induced vascular calcification (22). So far, data on pharmacologic augmentation of autophagy to improve uremic vascular media calcification *in vivo* are scarce. Recently, Peng and coworkers showed that autophagy plays a role in the calcification process of the aorta, but they were using a non-CKD calcification model (23).

We now aimed to characterize autophagy and its role in uremic media calcification induced by high-phosphate levels *in vivo* and *in vitro*. Furthermore, we influenced autophagy pharmacologically and studied the effects on uremic media calcification.

## Materials and Methods

### *In Vivo* Studies

Female 8-week-old dilute brown non-agouti 2 (DBA/2NCrl) mice were obtained from Charles River (Sulzfeld, Germany) and housed in a virus/antibody-free environment in the laboratory animal facility of the Medical University of Graz. These mice are susceptible to ectopic renal calcification and media calcification when exposed to increased oral phosphate loads (15).

To induce media calcification, they were placed on HPD (Altromin, Germany) containing 20.2 g/kg of phosphorus, 9.4 g/kg of calcium, 0.7 g/kg of magnesium, and 500 IU/kg of vitamin D3. The standard chow contained 7.0 g/kg of phosphorous, 10.0 g/kg of calcium, 2.2 g/kg of magnesium, and 1,000 IU/kg of vitamin D3. The mice were then followed for 5 and 12 days. For the interventional studies a rapamycin (LC Labs, Woburn, MA, USA) stock solution was prepared by dissolving rapamycin in 100% ethanol, which was then dissolved in sterile saline for intraperitoneal injection at a dose of 0.5 mg/kg body wt. Daily intraperitoneal rapamycin or vehicle administration was started either on 3 days prior or 5 days after starting HPD.

All animal experiments were approved by the Committee of the Ethics of Animal Experiments of the Austrian Ministry (BMWFW-66.010/0061-WF/V/3b/2016). All experiments were conducted under strict adherence of the law of Austria.

### *In Vitro* Studies

Mouse vascular smooth muscle cells (MOVAS) were bought from the American Type Culture Collection (ATTC, Manassas, VA, USA). Cells were cultured in high glucose Dulbecco’s Modified Eagle’s Medium (ATTC) supplemented with 10% FCS (Gibco, Life Technology, Vienna, Austria) and an antibiotic mixture of 1% penicillin/streptomycin (Gibco) at 37°C in a humidified, 5% CO_2_ atmosphere. Cells were seeded in six-well plates at a density of 1.0 × 10^4^ cells/cm^2^. At confluence, the medium was supplemented with either 1.25 mM β-glycerophosphate (βGP) (Sigma Aldrich, St. Louis, MO, USA) and 25 µg/mL ascorbic acid (AA) (Sigma Aldrich) or with 2.5 mM βGP and 50 µg/mL AA for 7, 14, and 21 days to induce calcification (24).

To induce or inhibit autophagy, cells were exposed to 10 µM rapamycin (LC labs), to 5 mM 3-methyladenine (3-MA) (Sigma Aldrich) or to 20 nM bafilomycin A1 (Sigma Aldrich). The medium including rapamycin, 3-MA, or bafilomycin was changed every other day.

To measure the autophagic flux on day 21, cells were exposed to 50 nM bafilomycin A1 for 3 h before harvesting.

### Reverse Transcription Real-Time Polymerase Chain Reaction

Murine tissue and cells were stored at −80°C and the AllPrep^®^DNA/RNA/Protein Mini Kit (Qiagen, Venlo, Netherlands) was used to isolate RNA, strictly following the manufacturer’s instructions. Complementary DNA transcripts from RNA was synthesized by using Superscript III Transcription Kit (Invitrogen, Carlsbad, CA, USA) and random primers (Invitrogen) for reverse transcription of 100 ng total RNA. Real-time PCR was performed in duplicates on a CFX96 Real-Time System (BioRad, Hercules, CA, USA).

For quantification of respective genes, TaqMan gene expression assays (Applied Biosystems, Foster City, DA, USA) for *Trp53in* (Mm00458142_g1), *Igfbp3* (Mm01187817_m1), *Hmox1* (Mm00516005-m1), *Adrb2* (Mm02524224_s1), *Atg16l1* (Mm00513085_m1), *Il-1beta* (Mm00434228_m1), *Tnf-alpha* (Mm00443258_m1), *Tbx21* (Mm00450960_m1), *Il-6* (Mm00446190_m1), *Foxp3* (Mm00475162_m1), and *Sm22-*α (Mm00441661_g1) were used. SYBR Green Mastermix (BioRad) was used for the detection of *Hprt* and *Runx2* with the following primers: *Hprt* forward 5′GCT TCC TCC TCA GAC CGC TTT TTG C 3′ and reverse 5′ATC GCT AAT CAC GAC GCT GGG ACT G 3′. *Runx2* forward 5′TCC TAT CTG AGC CAG ATG ACA TCC 3′ and reverse 5′CCG GTC TCC CCC GGG TAC C 3′. *Hprt* gene served as the housekeeping reference. Results were calculated with the 2ΔΔCT method.

### Western Blot Analysis

Protein was isolated by sonicating murine tissue as well as cells in a homogenization buffer (0.25 mol/L sucrose, 10 mmol/L HEPES, pH 7.5, and 1 mmol/L EDTA, pH 8.0) containing HALTTM Protease Inhibitor Cocktail, EDTA-free (Thermo Fisher Scientific, Rockford, IL, USA) and quantified with PierceTM BCA Protein Assay Kit (Thermo Fisher Scientific) according to the manufactures’ instruction. Aliquots of total protein were separated using 12% sodium dodecyl sulfate polyacrylamide gel electrophoresis for 1.5–2 h at 100 V. Subsequently, proteins were transferred to polyvinylidene fluoride membranes (Merck Millipore, Burlington, MA, USA) for 90 min 150 mA. Membranes were blocked in 5% nonfat dry milk/TBST for 3 h at room temperature and subsequently incubated with primary antibodies against GAPDH (Cell Signalling, Cambridge, UK), p62 (Abcam, Cambridge, UK), and LC3 (Novus Biologicals, Littleton, CO, USA) overnight at 4°C, followed by the appropriate HRP-conjugated secondary antibody (Cell Signalling) for 1 h at room temperature. Protein signals were visualized using PierceTM ECL Western Blotting Substrate (Thermo Fisher Scientific) and a ChemiDoc System (BioRad). Densitometric analyses were performed using Image Lab software (BioRad).

### Biochemical Analyses and *In Vitro* Detection of Calcification

Calcium content of the aortas and the kidneys was determined using the Calcium Detection Assay Kit (Abcam) following the manufacturer’s instructions and normalized to tissue weight. To quantify the calcium levels in MOVAS, cells were decalcified with HCl and the calcium content in supernatants was determined using the Calcium Detection Assay Kit (Abcam) according to the manufacturer’s instruction. Total protein was quantified using the PierceTM BCA Protein Assay Kit (Thermo Scientific) following the manufacturer’s instructions. The calcium content was normalized to the protein content.

Blood urea nitrogen (BUN) levels were measured using a colorimetric detection kit (Thermo Fisher Scientific) following the manufacturer’s instructions.

Calcium deposition was evaluated by staining the cells with Alizarin Red (Sigma Aldrich). Cells were washed with PBS, fixed in 4% paraformaldehyde for 15 min, stained with 2% Alizarin Red for 10 min at room temperature, and rinsed with distilled water. The stained cells were extracted with 10% cetylpyridium chloride (Sigma Aldrich) for 10 min. The OD was measured at 570 nm.

### Histological Evaluation

Aortas of DBA/2 mice were isolated and conserved for paraffin embedding. The extent of media calcification was determined histologically using Alizarin Red S staining. Alizarin Red staining was performed by incubating rehydrated paraffin sections in 2% Alizarin Red S solution (Sigma Aldrich) followed by rinsing in acetone and acetone xylene.

Light chain 3 was stained on paraffin sections using the three-layer immunoperoxidase staining protocol. Briefly, deparaffinized tissue sections were treated with standardized heat-mediated antigen retrieval in an automated de-cloaking chamber (Aptum, Southampton, UK), quenched in 0.3% H_2_O_2_ in methanol, blocked with biotin/avidin blocking kit (Vector Laboratories Inc., Burlingame, CA, USA), and stained with rabbit-derived primary antibody for LC3 (Novus Biologicals). A biotin-conjugated goat anti-rabbit IgG (Jackson ImmunoResearch Laboratories, West Grove, PA, USA) was used as a secondary antibody.

For performing nitro blue tetrazolium (NBT) staining, paraffin embedded sections were deparaffinized, washed in HBSS and incubated with NBT (1.6 mg/mL) in HBSS at 37°C for 20 min. Photographs were taken using a 20× objective.

Formalin-fixed renal tissue was embedded in paraffin and cut in 4 µm sections. The sections were stained with periodic acid Schiff’s.

### Immunofluorescence

MOVAS were grown on glass chamber slides and fixed in 4% paraformaldehyde for 20 min. After permeabilization with 100% ice-cold methanol, the cells were rinsed with PBS and washing buffer (0.1% BSA/PBS) and incubated for 45 min with blocking buffer. Then the slides were incubated for 1 h with the primary antibody rabbit-anti-α-smooth muscle actin (SMA) (Sigma Aldrich). The slides were extensively washed and incubated with FITC conjugated secondary antibody for 1 h at room temperature. Slides were mounted in ProLong Gold anti-fade with DAPI (Thermo Fisher Scientific) for imaging. Evaluation was performed on an LSM510 META (Zeiss, Oberkochen, Germany).

### Statistical Analyses

All statistical analyses were performed using GraphPad Prism 6.0 (GraphPad Software, La Jolla, CA, USA) and results are shown as mean ± SEM. Testing for normality was done using the Kolmogorov–Smirnov test with Dallal–Wilkinson–Lillifors correction. When comparing two groups, according to the distribution nonparametric Mann–Whitney *U* test or unpaired Student’s *t*-test was used. When comparing three or more groups, ANOVA or Kruskal–Wallis test was performed with subsequent Dunn’s test with adjustment for multiple comparisons. The survival comparison of rapamycin and vehicle-treated mice were plotted using the Kaplan–Meier method and log-rank (Mantel-Cox) test. A two-sided *p* < 0.05 was considered statistically significant.

## Results

### Induction of Autophagy in Aortic VSMC in an *In Vivo* Model of Uremic Media Calcification

**Figure 1:**
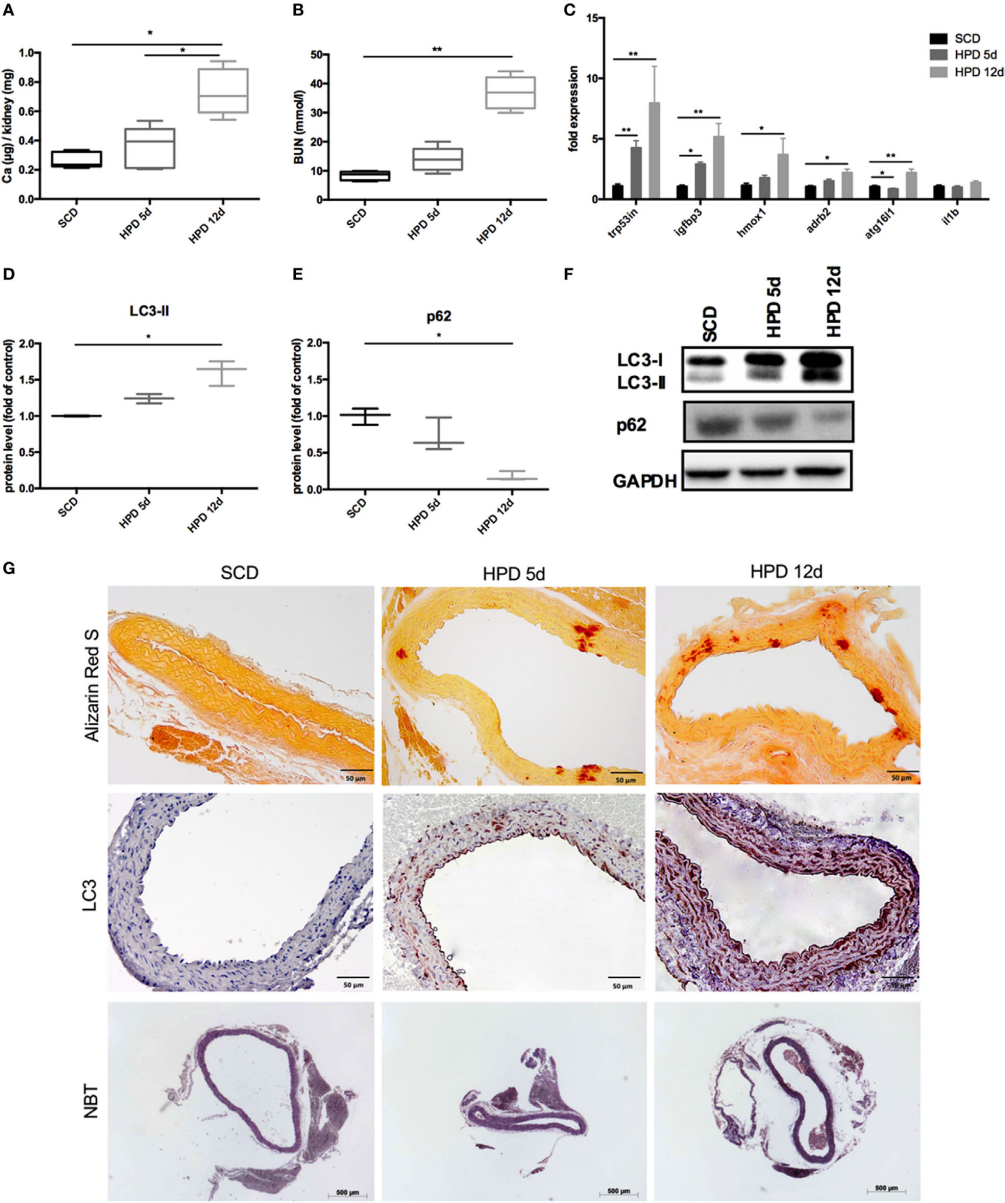
Autophagy is increased in uremic media calcification. DBA/2 mice were fed with high-phosphate diet (HPD; gray bars) or standard chow diet (SCD; black bars) for 5 or 12 days, respectively (SCD: *n* = 11; HPD 5d: *n* = 6, HPD 12d *n* = 4). **(A)** Calcium content in kidneys was measured quantitatively. **(B)** Blood urea nitrogen (BUN) levels were evaluated. **(C)** qPCR analysis of respective autophagy related genes and *Il-1beta* was performed from aortas. Western Blot analysis for **(D)** light chain 3 (LC3) and **(E)** p62 levels was performed. Three independent experiments have been performed. **(F)** A representative western blot is provided. **(G)** Aortas of SCD- and HPD-fed mice were stained with Alizarin Red, with an antibody detecting LC3 or nitro blue tetrazolium (NBT). Representative pictures from aorta sections are shown. All data are presented as mean ± SEM. *p < 0.05, **p < 0.01.

DBA/2 mice develop uremic media calcification when treated with HPD (13, 14). This was confirmed in our mice by measuring the calcium content in the kidney quantitatively (Figure 1A) and by evaluating BUN levels (Figure 1B) 5 and 12 days after starting HPD as compared to standard chow diet (SCD)-treated mice. By performing gene chip arrays, we previously detected autophagy pathway-associated genes to be regulated in DBA/2 mice that were subjected to HPD (14). We already showed an increase of *Trp53* and *Igfbp3* mRNA in aortas of HPD mice after 5 and 14 days (14). Therefore, we further evaluated DBA/2 mice for calcification and induction of autophagy in the aorta. We confirmed the increase in the autophagy-associated genes *Trp53in* and *Igfbp3* in aortas of 5 days HPD-treated mice as compared to SCD-treated controls, which was even more pronounced after 12 days of HPD treatment (Figure 1C). Also other autophagy-associated genes such as *Hmox1, Adrb2*, and *Atg16l1* showed a comparable pattern of regulation (Figure 1C). Of note, *IL-1beta* mRNA expression in aortas was not regulated at indicated time points (Figure 1C). Protein markers of autophagy, namely LC3-II and p62, were regulated accordingly to gene expression data implicating activation of autophagy in the aorta of HPD-treated mice (Figures 1D–F). By performing Alizarin Red S staining calcification of the aorta was located to the *Tunica media* and correlated with the length of HPD treatment (Figure 1G). In parallel, autophagy as detected by LC3-II immunohistochemistry was found to increase in vascular smooth cells of the *Tunica media* as well as in the vascular endothelial cell layer (Figure 1G). Furthermore, we performed NBT staining to detect ROS production in aortic specimen. An increased staining pattern in the *Tunica media* was detected in 12 days treated HPD mice as compared to SCD mice (Figure 1G).

### Induction of Autophagy in Mouse Vascular Smooth Muscle Cells (MOVAS) Under Calcifying Conditions

**Figure 2:**
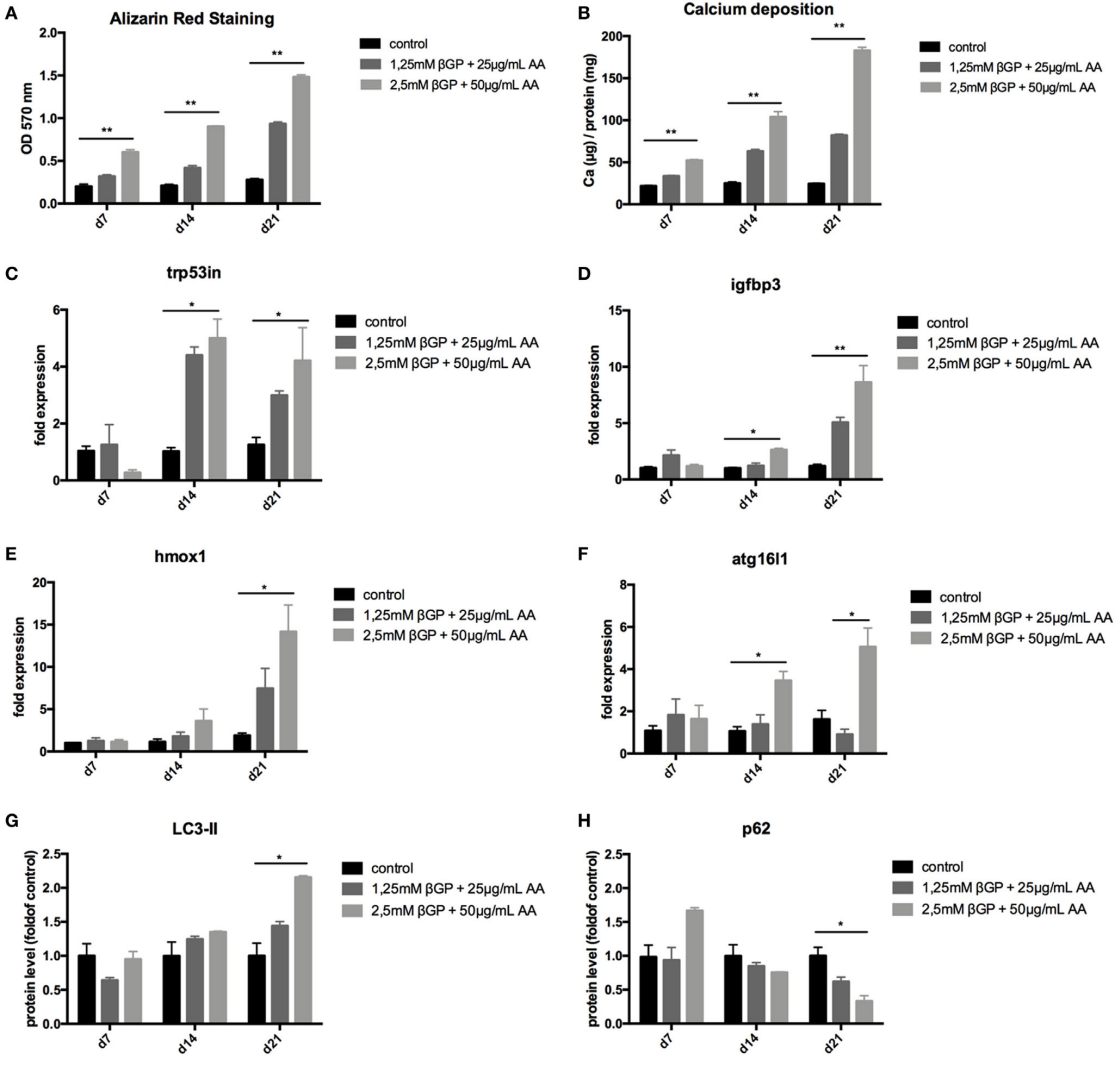

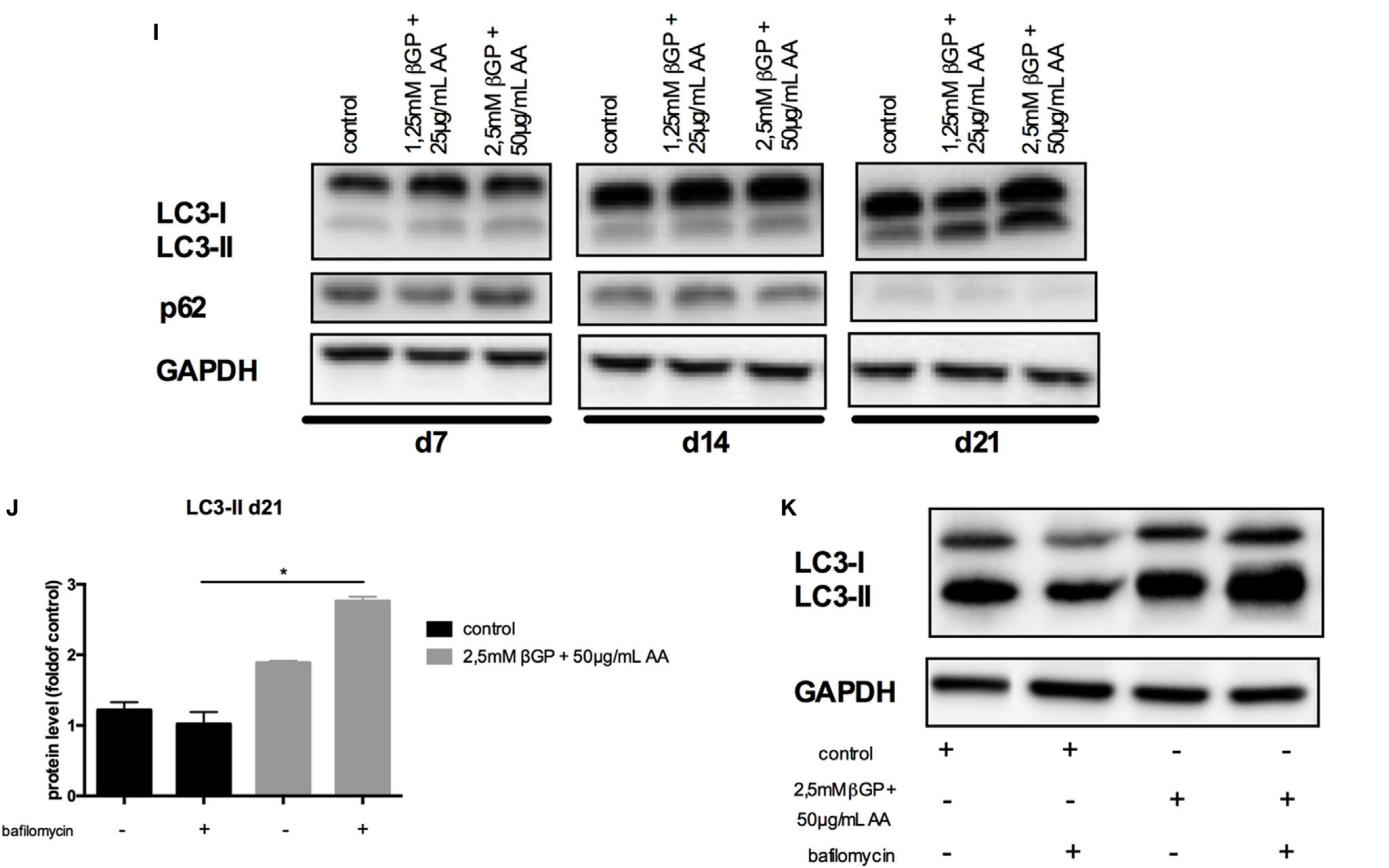
Calcification induces autophagy in MOVAS. MOVAS were cultured in the absence (black bars) or the presence (gray bars) of two different calcifying conditions for 7, 14, or 21 days (*n* = 4 per time point and group). **(A)** Calcium deposition in cells was analyzed by Alizarin staining and photometric quantification. **(B)** The cellular calcium content was evaluated quantitatively. **(C–F)** qPCR analysis for respective autophagy-associated genes was performed. Western Blot analysis for detection of **(G)** light chain 3 (LC3) and **(H)** p62 levels was performed. Three independent experiments were performed. **(I)** A representative western blot is shown. **(J,K)** After 21 days of culture, the autophagic flux was evaluated in MOVAS after short-term bafilomycin treatment (*n* = 3). **(K)** A representative LC3 Western blot is shown. All data are presented as mean ± SEM. *p < 0.05, **p < 0.01.

To mimic vascular calcification in smooth muscle cells *in vitro*, cells were subjected to calcifying conditions by supplementing the medium with two different concentrations of AA and β-glycerolphosphate for up to 21 days. These conditions resulted in increased calcification of MOVAS both over time and with increasing phosphate and AA supplementation as shown by quantification of Alizarin Red S stains (Figure 2A) and of the cellular calcium content (Figure 2B). Starting from day 14, autophagy-associated genes *Trp53in, Igfbp3, Hmox1*, and *Atg16l1* were increased in MOVAS treated with phosphate supplementation, reaching significance in the high-phosphate supplementation group (Figures 2C–F). The protein marker of autophagy, LC3-II, significantly increased in MOVAS treated for 21 days with high-phosphate supplementation (Figures 2G,I). In line, p62 decreased under the same conditions (Figures 2H,I). To evaluate the autophagic flux in our cells, MOVAS were treated with calcifying and non-calcifying conditions. After 21 days, they were subjected to bafilomycin for 3 h and subsequently analyzed for their LC3-II content. LC3-II was increased in MOVAS treated with bafilomycin as compared to respective controls proving that the calcifying medium increased autophagy in MOVAS (Figures 2J,K).

### Induction of Autophagy by Rapamycin-Treatment Decreased Calcification in MOVAS

**Figure 3:**
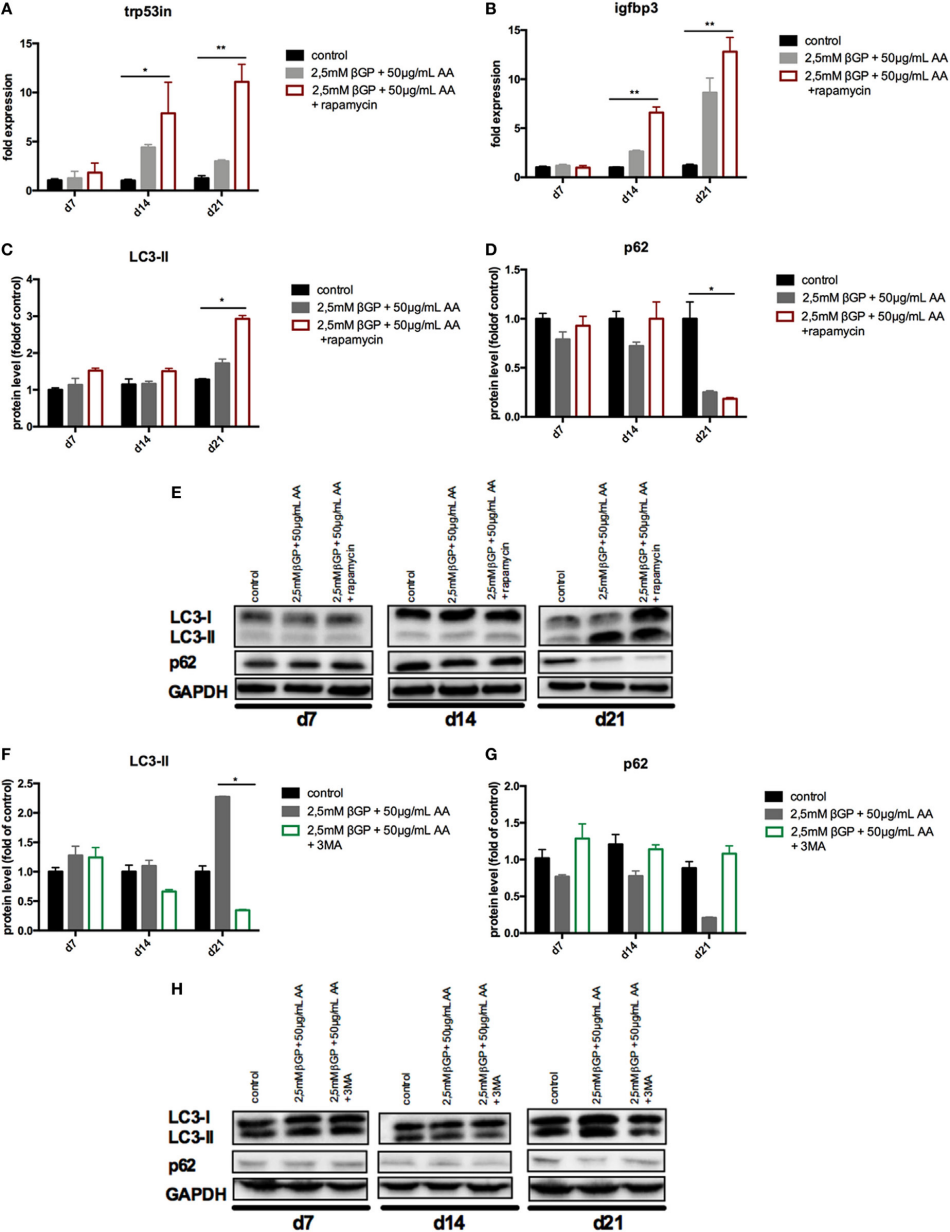
Autophagy is influenced in MOVAS by rapamycin and 3-methyladenine (3-MA). MOVAS were cultured in the presence (gray bars) or absence of calcifying conditions (black bars) and additionally exposed to 10 µM rapamycin (red bars) or to 5 mM 3-MA (green bars) for 7, 14, or 21 days. qPCR analysis [**(A,B)**; *n* = 4] from cells as well as Western Blot analysis [**(C–H)**; *n* = 3] were performed. Representative western blots for each treatment are shown **(E,H)**. All data are presented as mean ± SEM. *p < 0.05, **p < 0.01.

**Figure 4:**
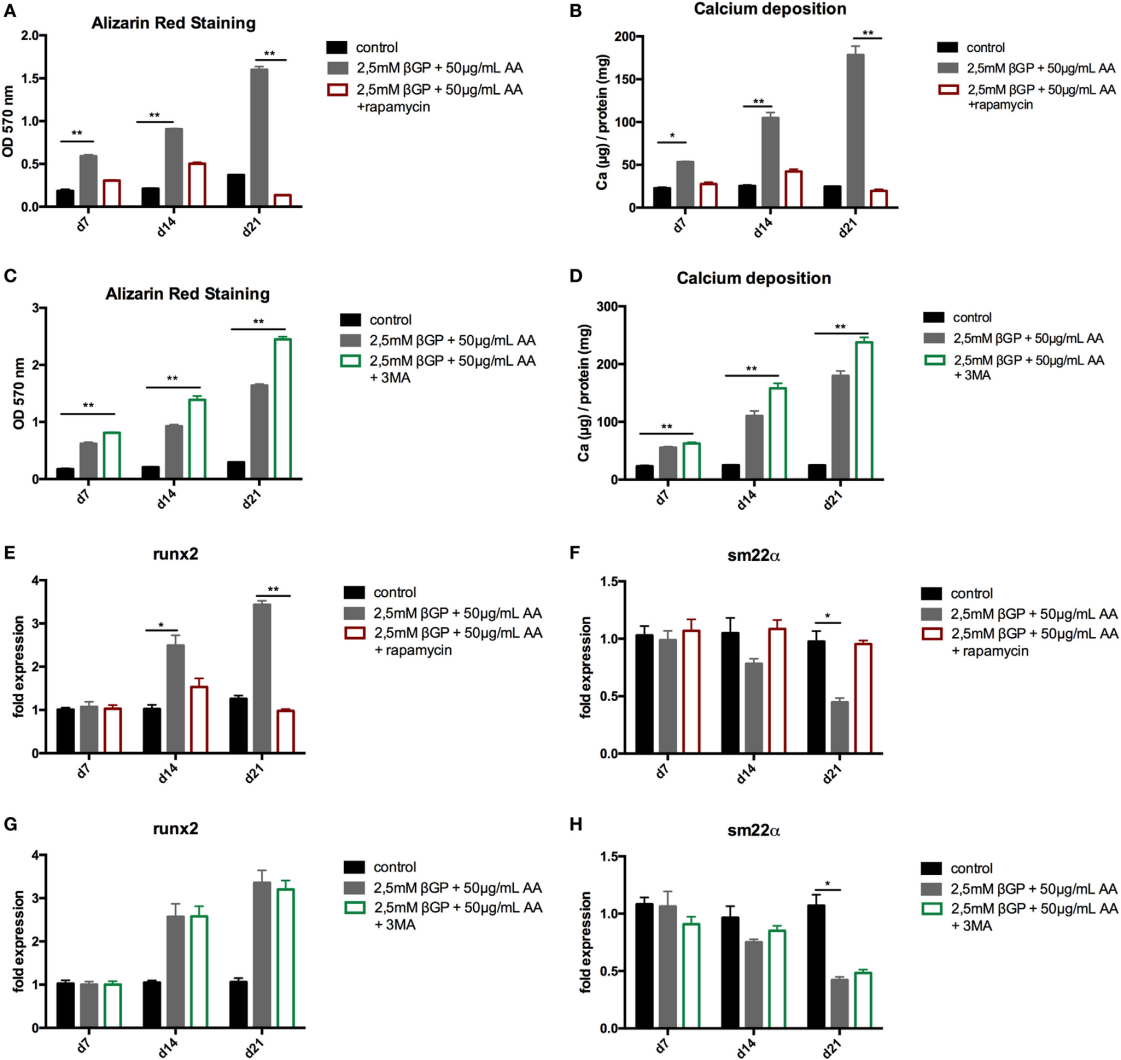
Induction and blockade of autophagy influences calcification of MOVAS. MOVAS were cultured in the presence (gray bars) or absence of calcifying conditions (black bars) and additionally exposed to 10 µM rapamycin (red bars) or to 5 mM 3-methyladenine (3-MA) (green bars) for 7, 14, or 21 days. Quantification of Alizarin Red S (*n* = 4) staining of cells treated either with rapamycin **(A)** or 3-MA **(C)** was done. Cells treated with rapamycin **(B)** or 3-MA **(D)** were analyzed for their calcium content. qPCR analysis [**(E–H)**; *n* = 4] from cells were performed. All data are presented as mean ± SEM. *p < 0.05, **p < 0.01.

**Figure 5:**
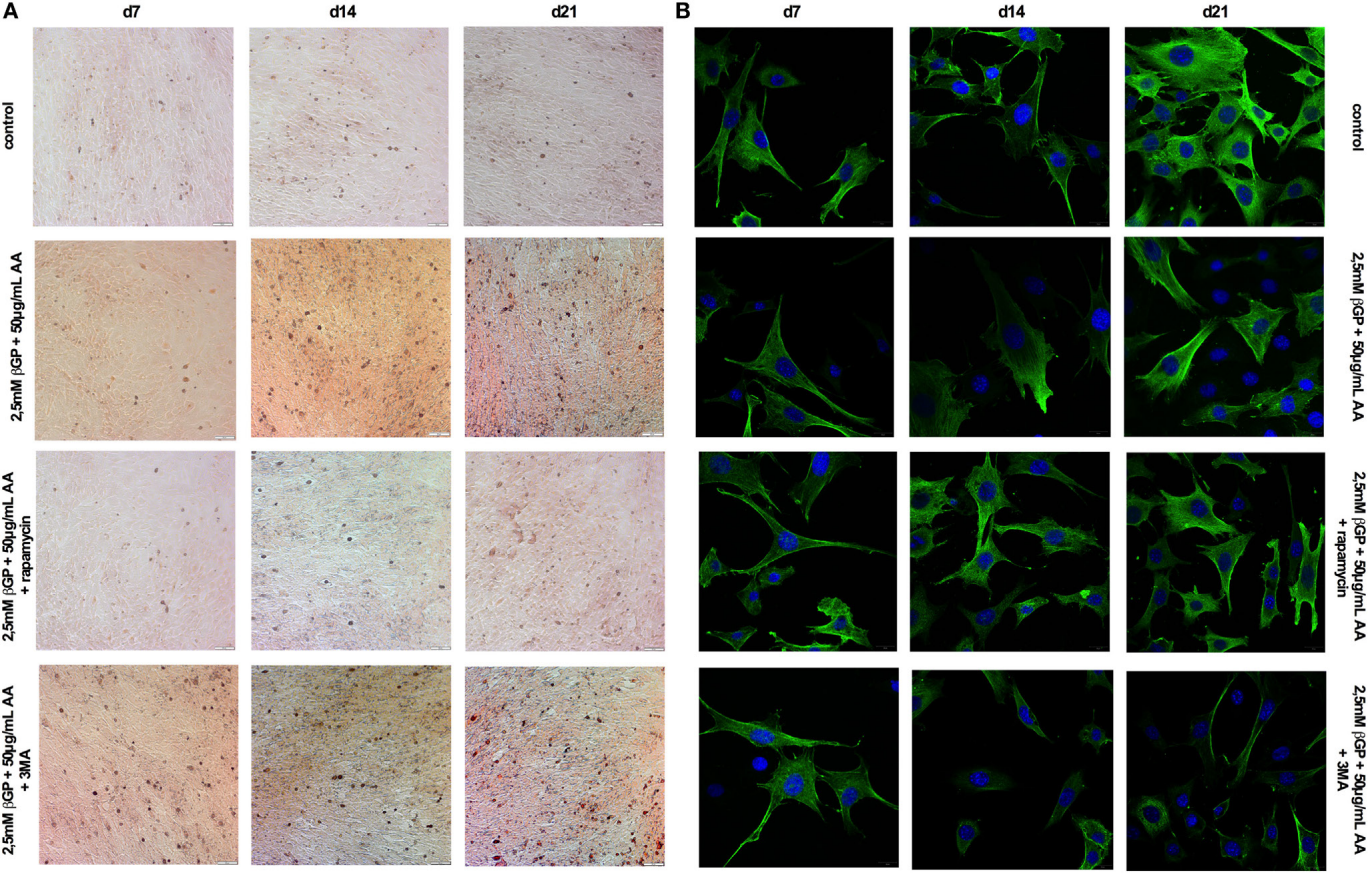
Induction and blockade of autophagy influences calcification and α-smooth muscle actin (SMA) expression of MOVAS. MOVAS were cultured in the presence or absence of calcifying conditions and additionally exposed to 10 µM rapamycin or to 5 mM 3-methyladenine (3-MA) for 7, 14, or 21 days. Representative pictures from **(A)** Alizarin Red S and **(B)** α-SMA stained cells are shown.

To influence autophagy in MOVAS, cells subjected to calcifying conditions (50 µg/mL AA and 2.5 mM β-glycerolphosphate) were treated with 10 µM rapamycin or 3-MA in order to increase or block autophagy, respectively. Rapamycin treatment nearly doubled the mRNA expression of the autophagy-associated genes *Trp53in* and *Igfbp3* after 14 and 21 days (Figures 3A,B). LC3-II protein was 1.5-fold increased in MOVAS under calcifying conditions as compared to control cells. Adding rapamycin to the calcifying medium resulted in a threefold increase in LC3-II protein on day 21 (Figures 3C,E). The level of p62 protein expression were comparable between MOVAS under calcifying conditions with or without rapamycin (Figures 3D,E). Nevertheless, a significant reduction of p62 protein in MOVAS was only detected under calcifying conditions with rapamycin as compared to MOVAS treated with control medium after 21 days of culture (Figures 3D,E). Even though rapamycin only moderately induced autophagy in MOVAS, calcification as measured by Alizarin stain was decreased by rapamycin already after 7 days of treatment. Significance was reached after 21 days of treatment (Figures 4A and 5A). This was confirmed by measuring the calcium content quantitatively in the cells (Figure 4B). *Runx2* mRNA expression, as a marker of osteoblastic VSMC differentiation, increased in MOVAS under calcifying conditions after 14 and 21 days (Figure 4E). This increase was blunted by rapamycin treatment (Figure 4E). *Sm22*α transcription as a marker for adult VSMCs was decreased in MOVAS under calcifying conditions after 21 days, which was abolished when cells were additionally treated with rapamycin (Figure 4F). Also α-SMA stainings as a marker for cellular contractility was found to be decreased in MOVAS after 14 and 21 days of calcifying conditions, which was blunted by rapamycin treatment (Figure 5B).

Next, MOVAS under calcifying conditions were treated with 3-MA, which blocks autophagy. After 21 days of treatment 3-MA reversed LC3-II and p62 protein levels to MOVAS incubated with standard medium (Figures 3F–H). Calcification of MOVAS increased by 3-MA treatment after 14 and 21 days (Figures 4C,D). 3-MA treatment did not differentially regulate *Runx2* and *Sm22*α transcription as compared to MOVAS under calcifying conditions (Figures 4G,H). α-SMA protein expression was further decreased in MOVAS treated with 3-MA after 14 and 21 days as compared to MOVAS under calcifying conditions (Figure 5B). Of note, we saw a similar regulation pattern when using an alternative autophagy blocker, namely bafilomycin (Figure S1 in Supplementary Material).

As control MOVAS incubated with standard medium were treated with either rapamycin, 3-MA, or bafilomycin, but no differences in the level of autophagy-associated genes and proteins was detected as compared to MOVAS incubated with standard medium (Figure S2 in Supplementary Material).

### Rapamycin-Treatment Improves Uremic Media Calcification and Survival *In Vivo*

**Figure 6:**
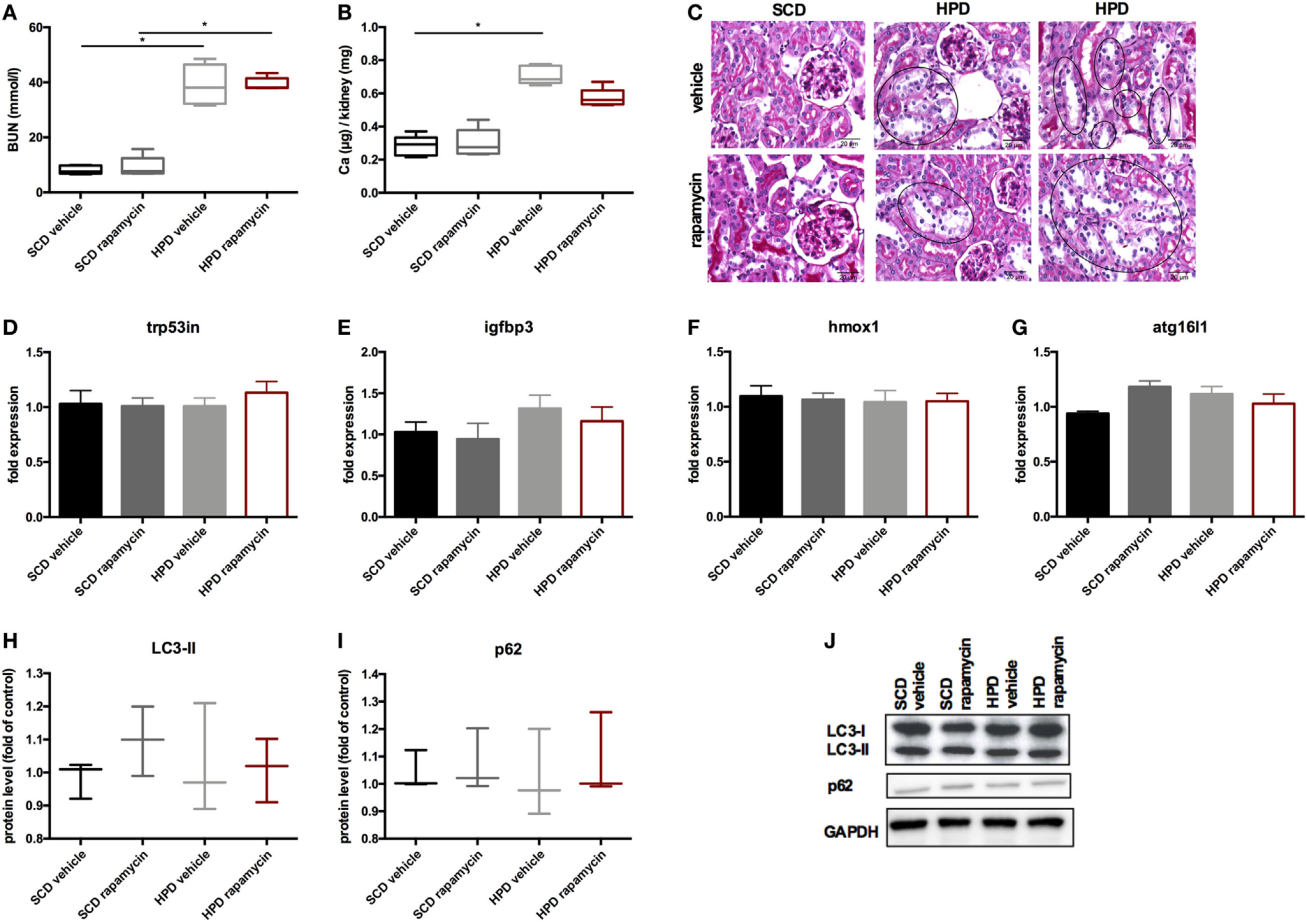
Rapamycin does not influence the renal phenotype. DBA/2 mice were fed with high-phosphate diet (HPD) or standard chow diet (SCD) for 12 days and were additionally treated with rapamycin or vehicle starting from day −3 (SCD + vehicle: black bar, SCD + Rapa: gray bar; HPD + vehicle: light gray bar; HPD + Rapa: red bar). **(A)** Serum-blood urea nitrogen (BUN) levels and **(B)** calcium content of the kidneys was evaluated in mice 12 days after starting HPD (*n* = 5). **(C)** Representative pictures of periodic acid Schiff’s stained kidney sections are provided. Areas of early necrotic tubular epithelial cells are marked by black circles. **(D–G)** qPCR analysis (*n* = 5 per group) for expression of respective autophagy-associated genes was performed from kidney tissue. **(H)** Light chain 3 (LC3) and **(I)** p62 levels were evaluated by Western Blot analysis (*n* = 3). A representative Western blot is shown **(J)**.

To increase autophagy *in vivo*, DBA/2 mice were treated with 0.5 mg/kg body weight of rapamycin or vehicle once daily starting 3 days before starting HPD. Controls were treated either with rapamycin or vehicle and were fed with SCD. First, mice were evaluated for the renal phenotype. DBA/2 mice fed with HPD displayed significantly increased BUN levels, which were not altered by rapamycin-treatment (Figure 6A). Also calcifications in the kidney did not significantly differ between HPD-fed mice treated with vehicle or rapamycin (Figure 6B). Kidney histology also remained unchanged in HPD mice treated either with vehicle or rapamycin (Figure 6C). HPD mice showed unaltered glomeruli, but signs of acute tubular injury mainly in the distal tubules. Distal tubular cells displayed vacuolization and loss of nuclear staining as markers for beginning cell necrosis (Figure 6C). Autophagy in the kidneys was not influenced by HPD or rapamycin treatment as shown by qPCR of autophagy-associated genes (Figures 6D–G) as well as LC3-II and p62 evaluations (Figures 6H–J).

**Figure 7:**
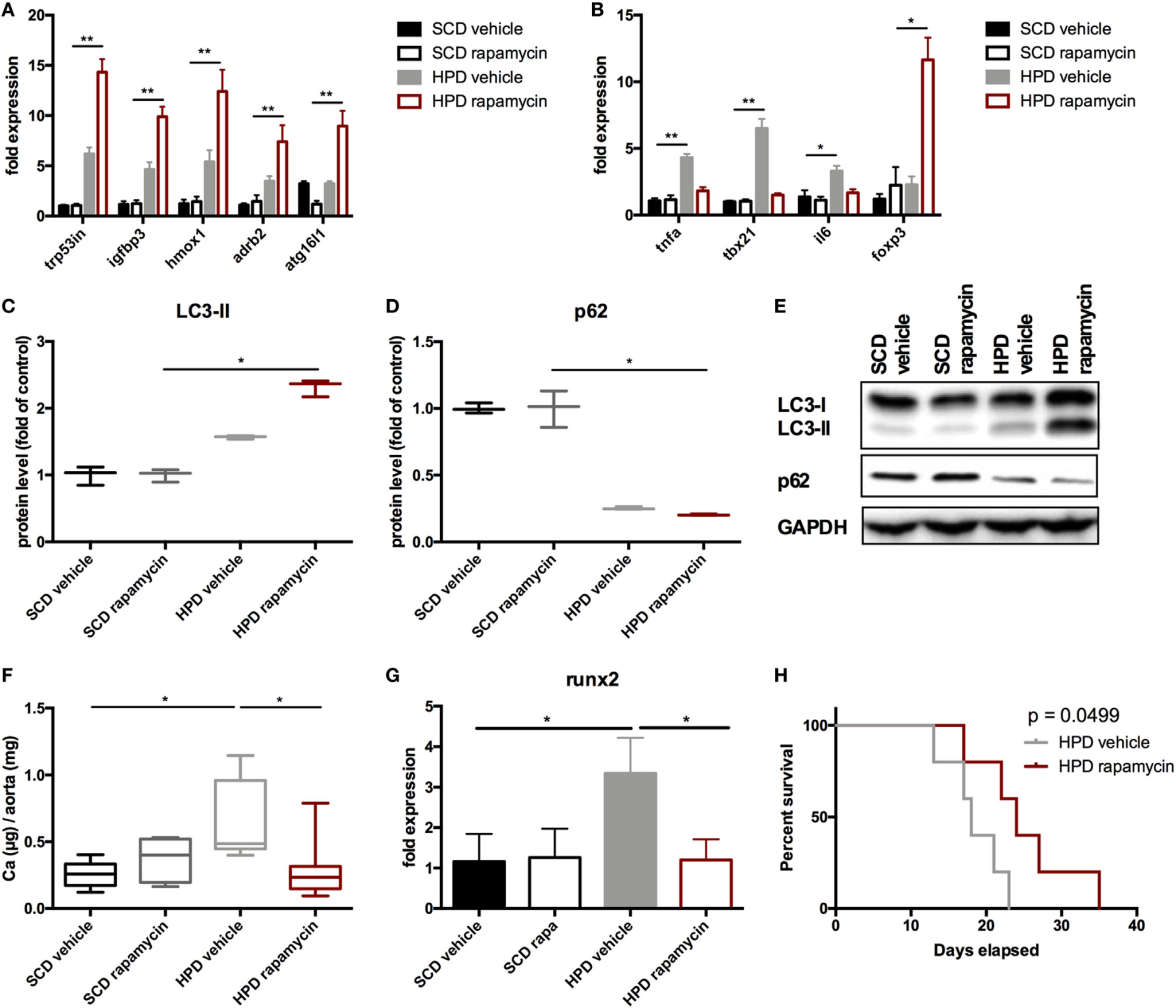
Rapamycin ameliorates uremic media calcification. DBA/2 mice were fed with high-phosphate diet (HPD) or standard chow diet (SCD) for 12 days and were additionally treated with rapamycin or vehicle starting from day −3 (SCD + vehicle: black bar, SCD + Rapa: gray bar; HPD + vehicle: light gray bar; HPD + Rapa: red bar). qPCR analysis (*n* = 5 per group) for expression of **(A)** respective autophagy and **(B)** inflammatory genes was performed from aortic tissue. **(C)** Light chain 3 (LC3) and **(D)** p62 levels were evaluated by Western Blot analysis (*n* = 3). A representative Western blot is shown **(E)**. **(F)** The total calcium content of aortas was analyzed (SCD: *n* = 5; HPD: *n* = 9). **(G)** qPCR analysis (*n* = 5 per group) for expression of Runx2 in aortas was performed. **(H)** A Kaplan–Meier-plot of mice subjected to HPD and treated with either Rapamycin (red line) or vehicle (gray line) is provided (*n* = 14 per group). All data are presented as mean ± SEM. *p < 0.05, **p < 0.01.

Next, the vascular phenotype in aortas was evaluated. Autophagy was increased in HPD mice and rapamycin-treatment even augmented this increase as shown in autophagy-associated gene expression (Figure 7A) as well as increase in LC3-II and decrease in p62 protein levels (Figures 7C–E). Aortic calcification as shown by calcium contents in the aorta was significantly reduced in rapamycin-treated as compared to vehicle HPD mice (Figure 7F). Rapamycin-treatment blunted the increase of pro-inflammatory cytokine mRNA expression such as *Tnf-alpha* and *Il-6* in aortas of HPD mice (Figure 7B). In line, *tbx21* mRNA expression, which transcribes the master gene regulator of TH1 cells Tbet, was decreased by rapamycin-treatment in aortas of HPD-fed mice (Figure 7B). The master gene regulator of regulatory T cells *foxp3* was significantly increased in aortas of rapamycin-treated HPD mice (Figure 7B). Aortas of HPD mice showed a significant increase in the transcription of *Runx2*, which was reduced to the level found in aortas of SCD mice when HPD mice were treated with rapamycin (Figure 7G). Previously, we provided evidence that DBA/2 mice on HPD die because of bradycardia and sudden cardiac death due to extensive cardiovascular calcification (13). Thus, we evaluated whether rapamycin-treatment improves survival in HPD-fed mice. HPD-mice treated with rapamycin survived significantly longer when treated with rapamycin (Figure 7H).

### Rapamycin-Treatment Delays Progression of Established Uremic Media Calcification

**Figure 8:**
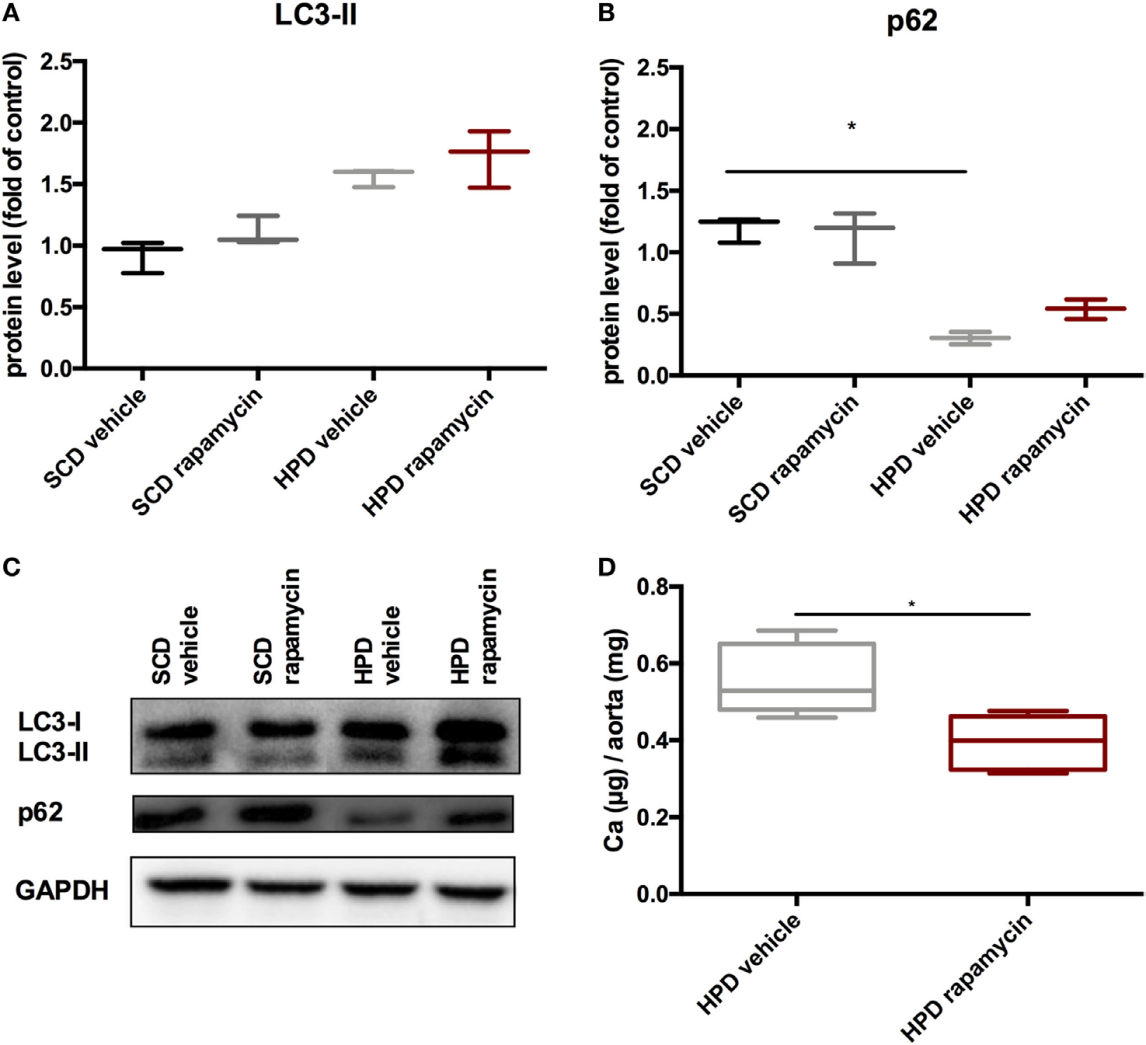
Rapamycin improves uremic media calcification in mice with established vascular calcification. DBA/2 mice were fed with high-phosphate diet (HPD) or standard chow diet (SCD) for 12 days and were additionally treated with Rapamycin or vehicle starting from day 5 (SCD + vehicle: black bar, SCD + Rapa: gray bar; HPD + vehicle: light gray bar; HPD + Rapa: red bar). Western Blot analysis (*n* = 3) for detection of **(A)** light chain 3 (LC3) and **(B)** p62 levels were performed. **(C)** A representative Western blot is shown. **(D)** Total calcium content of aortas was evaluated (*n* = 5 per group). All data are presented as mean ± SEM. *p < 0.05.

To test whether rapamycin has the capacity to improve uremic media calcification when started with established vascular calcification, rapamycin-treatment was started on day 5 of HPD treatment and followed until day 12. Here, rapamycin-treatment did not significantly influence LC3-II and p62 protein expression in aortas of HPD-fed mice (Figures 8A–C). Nevertheless, calcification of aortas was still significantly decreased in rapamycin- as compared to vehicle-treated HPD-fed mice (Figure 8D).

## Discussion

Here, we provide compelling evidence that uremic media calcification increases autophagy in VSMC *in vitro* and *in vivo*. Since pharmacologically enhancing autophagy resulted in improved uremic media calcification and survival, autophagy provides an endogenous protective response.

There exist increasing data that autophagy is a protective response of the body to counteract atherosclerosis, but those models mainly used Apo E knockout mice thereby reflecting human atherosclerosis rather than uremic media calcification (21). The data on autophagy in uremic media calcification, which critically differs from atherosclerosis in terms of histological features and pathomechanisms (25), are scarce. Dai and coworkers provided compelling *in vitro* evidence that stimulation of VSMC by high-phosphate conditions increases autophagy in cells and protects from calcification (22). We on the one hand confirmed their *in vitro* findings and on the other hand extended their findings to the *in vivo* situation. We used an acute model of uremia induced by HPD, which is accompanied by vascular media calcification (14). Here, calcification of the *Tunica media* was paralleled by an increase of autophagy in VSMC. When increasing autophagy by using rapamycin in our high-phosphate fed DBA/2 mice, we were not only able to significantly decrease calcification of aortas but also to extend the life span of mice. In our hands, rapamycin was also able to decrease calcification of the aortas when started with already established vascular calcification. Importantly, the protective effects of rapamycin were not attributable to an improved kidney function since neither renal function nor calcification was altered by rapamycin treatment. So far, we can only speculate about the different susceptibility to protection of calcification by autophagy induction in aortas versus kidney. Probably the calcification process in the kidney is overwhelming in our model thereby resulting in necrosis rather than autophagy.

A major limitation of our *in vivo* studies is the fact that the systemic anti-inflammatory effects of rapamycin may contribute to the improved outcome of rapamycin-treated mice. Rapamycin is a well-known inducer of regulatory T cells *in vivo* and *in vitro* (26, 27). In line, *Foxp3* transcription was significantly increased in aortic samples of rapamycin-treated mice while macrophage and Th1 cell marker transcription were suppressed. Still, the role of inflammation in the development of uremic media calcification is heavily discussed (14, 25, 28). Our group recently proved evidence that infiltration of immune cells in our model of acute uremic media calcification is rather a secondary phenomenon induced by calcification (14). Future *in vivo* experiments using autophagy inducers, which are more specific and do not influence the immune system such as TAT-Beclin (29), are clearly needed to show the impact of autophagy on calcification of the aorta and survival of mice.

The classical way to induce autophagy in cells is *via* nutrient/amino acid deprivation as seen in fasting (21), which is also a plausible inducer in our model since food intake and body weight drop significantly on HPD (data not shown). Furthermore, the production of ROS induces autophagy. This activation pathway has been attributed to phosphate-induced autophagy in VSMC in the past (22) and we also found *in vivo* signs of increased ROS production in aortas induced by HPD, which were shown by NBT stains. Still, further experiments are needed to delineate the role of ROS in our *in vivo* setting. Recent evidence suggests that activation of the inflammasome increases autophagy as well. Obviously, there exists a two-way regulation since autophagy also leads to removal of intracellular DAMPs, inflammasome components, or cytokines resulting in decreased inflammasome activation (30). The autophagy related gene *Atg16l1* has been linked to the inflammasome in the past since depletion of this gene resulted in an enhanced endotoxin-induced IL-1beta production (31). We found *Atg16l1* mRNA transcripts to be significantly increased in aortic samples of HPD fed mice, but we did not detect changes in *IL-1beta* transcription levels in aortic samples of high-phosphate treated mice. Thus, this pathway might not be critically involved in the calcification process of the aorta. Most of the autophagy activation pathways have been proven to be mTOR-dependent, but also independent pathways have been described (21).

It is generally believed that autophagy releases amino acids and fatty acids to produce energy for survival. However, if cellular damage becomes irreparable, cells can destroy themselves completely by autophagy (32). So far, it has been proposed that autophagy inhibits the formation of matrix vesicle release in VSMC, which is crucial for the calcification process of the cells (22). Nevertheless, whether this process also holds true in the *in vivo* situation needs further exploration. Contrary to Dai et al. (22), we detected *Runx2* mRNA transcription—as a marker of osteogenic transdifferentiation of VSMC (33)—to be significantly increased under calcifying conditions *in vivo* and *in vitro*. Interestingly, rapamycin-treatment blunted the *Runx2* mRNA increase both in MOVAS and in the aorta of HPD-fed mice. In line with our hypothesis that autophagy inhibits osteogenic transdifferentiation of VSMCs, α-SMA protein expression and *SM22*α transcription were found to be increased in rapamycin-treated MOVAS, whereas 3-MA-treated MOVAS showed decreased expression of both markers. Reduction of both α-SMA and SM22α has been attributed to osteogenic transdifferentiation of VSMCs (34, 35).

In summary, autophagy is an endogenous protective response of VSMC during uremia to prevent calcification possibly by inhibiting osteogenic transdifferentiation of VSMCs. Increasing autophagy may be an attractive strategy to improve vascular calcification in the CKD population.

## Ethics Statement

All animal experiments were approved by the Committee of the Ethics of Animal Experiments of the Austrian Ministry (BMWFW-66.010/0061-WF/V/3b/2016). All experiments were conducted under strict adherence of the law of Austria.

## Author Contributions

BF—performed experiments, analyzed data, and drafted the manuscript. AK, DD, and PE—designed study and revised manuscript. CS, KeS, and MK—performed experiments and revised manuscript. MP and KaS—performed experiments, analyzed data, and revised manuscript. AR—revised manuscript. KE—designed study, analyzed data, and drafted the manuscript.

## Conflict of Interest Statement

The authors declare that the research was conducted in the absence of any commercial or financial relationships that could be construed as a potential conflict of interest.

## Supplementary Material

Figure S1 Autophagy and calcification is influenced in MOVAS by bafilomycin. MOVAS were cultured in the presence (gray bars) or absence of calcifying conditions (black bars) and additionally exposed to 20 nM bafilomycin (dashed green bars) for 7, 14, or 21 days (*n* = 4 per group). Quantification of **(A)** Alizarin Red S staining of cells and **(B)** calcium deposition in MOVAS was done. **(C,D)** qPCR as well as **(E–G)** Western Blot analysis from MOVAS were performed. Representative western blots are shown **(G)**. All data are presented as mean ± SEM. **p* < 0.05, ***p* < 0.01.

Figure S2 Calcification and autophagy remained unaltered in MOVAS under non-calcifying conditions by rapamycin, 3-methyladenine (3-MA), and bafilomycin. MOVAS were cultured in the absence of calcifying conditions (black bars) and additionally exposed to 10 µM rapamycin (red bars) or to 5 mM 3-MA (green bars) or to 20 nM bafilomycin (dashed green bars) for 7, 14, or 21 days (*n* = 4 per group). Quantification of **(A)** Alizarin Red S staining of cells and **(B)** calcium deposition in MOVAS was done. **(C–F)** qPCR as well as **(G–L)** Western Blot analysis from MOVAS were performed. Representative western blots are shown **(I,L)**. All data are presented as mean ± SEM.
