# Supplementary figures and images for: Autophagy Protects From Uremic Vascular Media Calcification
Source: Front Immunol. 2018 Aug 14;9:1866. doi: 10.3389/fimmu.2018.01866 (PMC6102358; doi:10.3389/fimmu.2018.01866)

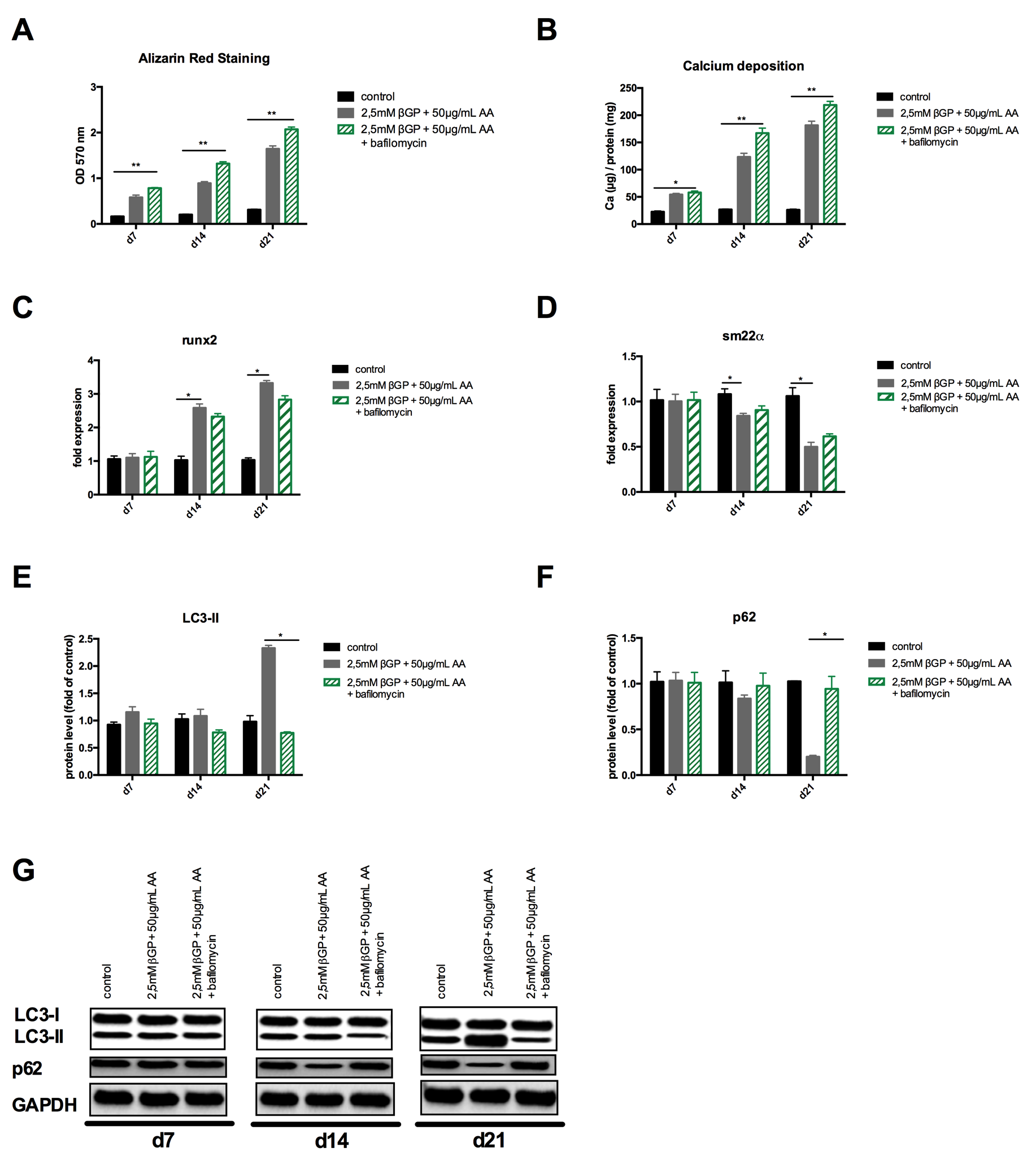

Supplement: Figure S1 — Autophagy and calcification is influenced in MOVAS by bafilomycin. MOVAS were cultured in the presence (gray bars) or absence of calcifying conditions (black bars) and additionally exposed to 20 nM bafilomycin (dashed green bars) for 7, 14, or 21 days (n = 4 per group). Quantification of (A) Alizarin Red S staining of cells and (B) calcium deposition in MOVAS was done. (C,D) qPCR as well as (E–G) Western Blot analysis from MOVAS were performed. Representative western blots are shown (G). All data are presented as mean ± SEM. *p < 0.05, **p < 0.01. [file Image_1.tiff]

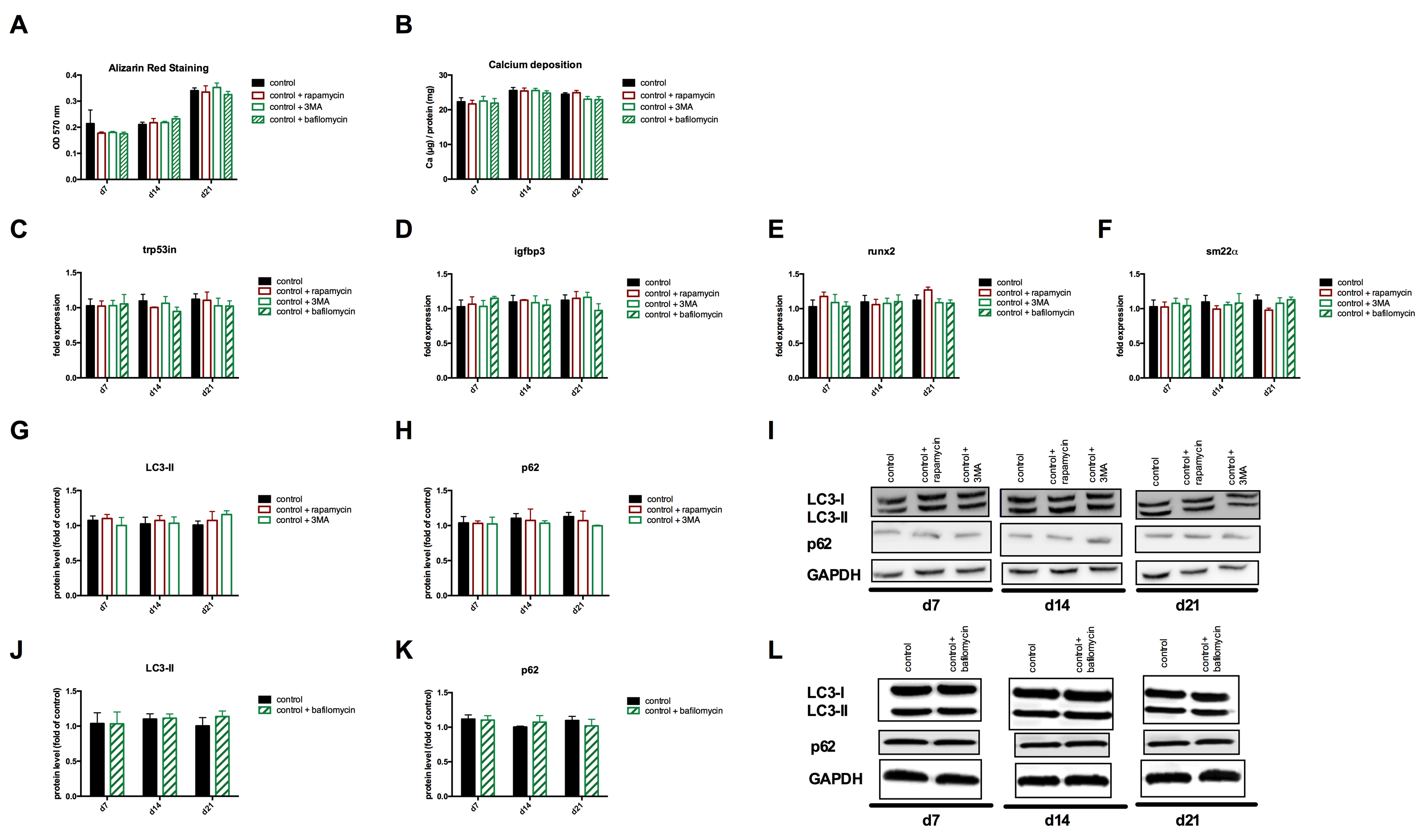

Supplement: Figure S2 — Calcification and autophagy remained unaltered in MOVAS under non-calcifying conditions by rapamycin, 3-methyladenine (3-MA), and bafilomycin. MOVAS were cultured in the absence of calcifying conditions (black bars) and additionally exposed to 10 µM rapamycin (red bars) or to 5 mM 3-MA (green bars) or to 20 nM bafilomycin (dashed green bars) for 7, 14, or 21 days (n = 4 per group). Quantification of (A) Alizarin Red S staining of cells and (B) calcium deposition in MOVAS was done. (C–F) qPCR as well as (G–L) Western Blot analysis from MOVAS were performed. Representative western blots are shown (I,L). All data are presented as mean ± SEM. [file Image_2.tiff]
